# Triglyceride to high density lipoprotein cholesterol ratio among adolescents is associated with adult hypertension: the Kangwha study

**DOI:** 10.1186/s12944-018-0861-y

**Published:** 2018-09-10

**Authors:** Hyungseon Yeom, Hyeon Chang Kim, Ju-Mi Lee, Yongwoo Jeon, Il Suh

**Affiliations:** Department of Preventive Medicine, Yonsei University College of Medicine, 50-1 Yonsei-ro, Seodaemun-gu, Seoul, 03722 Republic of Korea

**Keywords:** Triglyceride, High density lipoprotein cholesterol, Adolescent, Adulthood, Hypertension, List of abbreviations., TG: triglyceride; HDL-C: high density lipoprotein cholesterol; SBP: systolic blood pressure; DBP: diastolic blood pressure; BMI: body mass index.

## Abstract

**Background:**

The triglyceride to high density lipoprotein cholesterol (TG/HDL-C) ratio associated with hypertension in adults. However, whether the TG/HDL-C ratio in adolescents predicts future hypertension remains unclear. Here, we evaluated the prospective association between the TG/HDL-C ratio in adolescents and hypertension in early adulthood.

**Methods:**

The Kangwha Study is an ongoing prospective cohort study that has tracked the blood pressure of first grade elementary school students since 1986. We followed up 272 participants who completed health examinations at the age of 16 and 35 years. We excluded 27 participants with adolescent hypertension, defined as those whose blood pressures were above the age- and sex-specific 95th percentiles of the Korean population, and finally analysed 245 participants. We defined high and low TG/HDL-C ratio groups according to the age- and sex-specific 75th percentile of the TG/HDL-C ratio (1.04 for boys and 0.81 for girls) of the Korean population. Adult hypertension was defined by a systolic/diastolic blood pressure ≥ 140/90 mmHg or by taking antihypertensive medication at the age of 35 years. Logistic regression analysis was performed to evaluate the association between adolescent TG/HDL-C ratio and adult hypertension after adjusting for age at follow-up, sex, baseline systolic blood pressure, waist circumference, and total cholesterol and fasting glucose levels.

**Results:**

During the 20-year follow-up, 11 (18.3%) individuals developed hypertension in the high TG/HDL-C ratio group and 10 (5.4%) individuals developed hypertension in the low TG/HDL-C ratio group. The adjusted odds ratio for incident hypertension in the high TG/HDL-C ratio group, compared with the low TG/HDL-C ratio group, was 3.40 (95% confidence interval 1.24–9.31).

**Conclusions:**

High TG/HDL-C ratio in adolescence is associated with hypertension in early adulthood.

**Electronic supplementary material:**

The online version of this article (10.1186/s12944-018-0861-y) contains supplementary material, which is available to authorized users.

## Background

Dyslipidaemia, including elevated triglyceride (TG), elevated low-density lipoprotein cholesterol, and low high-density lipoprotein cholesterol (HDL-C), are independently associated with hypertension or other cardiometabolic risk factors [1–4]. The combinations of these lipids are also useful for predicting cardiovascular risks. Some studies have reported that the TG to HDL-C (TG/HDL-C) ratio is a useful predictor of hypertension [5–7].

In one study of 947 individuals, significantly higher values of both systolic and diastolic blood pressure were observed in the high TG/HDL-C ratio group, categorised by a cut-off value of ≥1.1 for women and ≥ 1.5 for men than in the low TG/HDL-C ratio group [5]. Another study of 1566 participants, which used the same cut-off points as above, also showed similar results [6]. One prospective study followed middle eastern women for over 6.4 years and found that an increase of one standard deviation in TG/HDL-C ratio increases the risk of incident hypertension by 18% [7]. The highest TG/HDL-C ratio quartile had a 70% higher risk of hypertension than the lowest quartile did.

Studies on an adolescent population have also obtained similar findings [8]. A cross-sectional study of 893 male subjects, aged 10–26 years, reported that the highest TG/HDL-C ratio tertile had the highest systolic and diastolic blood pressure [8]. The study also reported that several measurements of arterial stiffness, such as augmentation index and pulse wave velocity, were impaired in the highest TG/HDL-C ratio group. However, among these studies, none have prospectively evaluated the predictive value of the TG/HDL-C ratio for hypertension incidence at adulthood using an adolescent population. Thus, this study evaluated whether the TG/HDL-C ratio in adolescents could predict hypertension incidence in young adults.

## Methods

The Kangwha Study is an ongoing community-based prospective cohort study that started in 1986, with the purpose of tracking blood pressure and evaluating related determinants. The initial participants included 484 first-grade students from elementary schools in Kangwha island, most of whom were aged 6 years at enrolment. During annual follow-ups until high school graduation (year 1997), classmates of the initial participants were newly enrolled into the cohort. We followed up 742 individuals who participated in 1996 when blood tests were conducted until the latest adulthood follow-up health examination (2014–2017). Informed consent was obtained from all participants in our study. The study protocol was approved by the institutional review board of the Severance Hospital at Yonsei University Health System (4–2014-0914).

At the baseline examination in 1996, we measured the height, weight, waist circumference, and blood pressure and conducted blood tests for all the participants. Systolic blood pressure (SBP) and diastolic blood pressure (DBP) were measured from the participant’s right brachial artery with a standard mercury sphygmomanometer (Baumanometer, New York, USA) after a 5-min rest period in the sitting position. SBP was measured at the first Korotkoff sound, and DBP was measured at the fourth Korotkoff sound. We measured blood pressure at least two times per participant. Blood samples were collected after overnight fasting (at least ≥8 h). Total cholesterol, TG, and HDL-C levels were measured by enzymatic methods (Hitachi-747, Japan).

At the adulthood follow-up health examination during 2014 and 2017, we conducted health surveys and blood pressure measurements. Information on demographic characteristics, diagnosis of hypertension, and antihypertensive medication status were obtained through the questionnaire. Right arm blood pressure was measured after a rest of at least 5 min, in the sitting position, and the participants were asked to remain still and relax during the measurements. A cuff tailored to their individual mid-arm circumference was used, and trained research personnel conducted blood pressure measurements using an automated oscillometric device (HEM-7080, Omron Health, Matsusaka, Japan), in accordance with a standardised protocol. SBP and DBP were measured three times each, at 2-min intervals. In addition to those who visited our centre and received health examinations, we used surveys via mail and online for those who could not visit our centre. We obtained self-reported health status, which included diagnoses of hypertension, and personally-measured blood pressure.

As blood pressure was measured multiple times (twice during adolescence and thrice during adulthood follow-up), the average value was used for statistical analyses. Adolescent hypertension was defined as SBP/DBP higher than the sex and height specific 95th percentiles of the Korean population at age of 16 years (Additional file 1: Table S1). Adult hypertension was defined as SBP/DBP ≥140/90 mmHg or as taking antihypertensive medication at the follow-up examination. Written informed consent was obtained from each participant. Body mass index (BMI) was calculated as the weight in kilograms divided by the square of height in meters. The most commonly used cut-off point of TG/HDL-C ratio in previous studies was based on the highest 75th percentile in their study population. Therefore, we classified the participants into the high, and the low TG/HDL-C ratio groups according to the age, and sex-specific 75th percentile of the TG/HDL-C ratio in the Korean adolescent population [9]. The cut-off point of the TG/HDL-C ratio was 1.04 for boys and 0.81 for girls.

Continuous variables were expressed as means and standard deviation, and categorical variables were expressed as numbers with proportions. Student’s *t*-tests and chi-square tests were used to compare the baseline characteristics of participants according to participation in follow-ups, and to the TG/HDL-C ratio groups. Multiple linear logistic regression models were used to evaluate the association between TG/HDL-C ratio and adult hypertension, and the results were expressed as odds ratios and 95% confidence intervals. We used several regression models, including a crude model, and multi-adjusted models. Model 1 shows the unadjusted regression analysis between the TG/HDL-C ratio groups and adult hypertension. Model 2 was adjusted for sex and age at follow-up. Model 3 was adjusted for sex, age at follow-up, baseline SBP, waist circumference, total cholesterol, and fasting glucose. We conducted a sensitivity analysis, excluding participants who were followed up via mail or online. All analyses were performed with Statistical Analyses Software (SAS, version 9.4, SAS, Inc., Cary, NC, USA), and *p* < 0.05 were considered to indicate statistical significance.

## Results

Of the 742 participants who were enrolled at baseline in 1996, 256 participants underwent health examination follow-ups from 2014 to 2017, and 16 participants responded to the survey either online or by mail. We excluded 27 participants (25 participants who underwent health examination and 2 participants who responded online or by mail) who had adolescent hypertension. Finally, 245 participants were analysed to evaluate the association between the TG/HDL-C ratio in adolescence and hypertension in adulthood. Table 1 shows the baseline characteristics of the participants. The mean TG/HDL-C ratio was 1.17 (standard deviation 0.74).

**Table 1:** Baseline characteristics of the study population (245 participants)

Variables	Values
Height (cm)	165.4 ± 8.0
Weight (kg)	56.8 ± 8.5
Body mass index (kg/m^2^)	20.7 ± 2.5
Waist circumference (cm)	68.5 ± 6.3
Systolic blood pressure (mmHg)	113.9 ± 9.2
Diastolic blood pressure (mmHg)	70.9 ± 6.6
Total cholesterol (mmol/L)	3.93 ± 0.71
Triglyceride (mmol/L)	1.25 ± 0.59
High density lipoprotein cholesterol (mmol/L)	1.16 ± 0.26
Fasting glucose (mmol/L)	4.02 ± 0.61
TG/HDL-C ratio	1.17 ± 0.74
Sex	
Male	129 (52.6)
Female	116 (47.4)

*TG* triglyceride, *HDL-C* high density lipoprotein cholesterol

Values are presented as mean ± standard deviation, or numbers (%)

Table 2 shows the comparison of baseline characteristics of participants according to the high and low TG/HDL-C ratio groups. Among the 14 participants who had been evaluated by online or mail surveys, 7 were in the high TG/HDL-C ratio group and 7 were in the low TG/HDL-C ratio group. A higher mean waist circumference was observed in the high TG/HDL-C ratio group than in the low TG/HDL-C group (70.9 vs. 67.7 cm, *p* = 0.001). Differences in the other variables were not statistically significant.

**Table 2:** Baseline characteristics of the participants by adolescent TG/HDL-C ratio

	Low TG/HDL-C group^a^ (185)	High TG/HDL-C group (60)	*p*-value
Height (cm)	165.5 ± 7.8	165.8 ± 8.4	0.68
Weight (kg)	56.2 ± 7.5	59.0 ± 11.1	0.07
Body mass index (kg/m^2^)	20.5 ± 2.3	21.4 ± 3.1	0.06
Waist circumference (cm)	67.7 ± 5.4	70.9 ± 8.2	0.001
Systolic blood pressure (mmHg)	113.8 ± 9.19	114.3 ± 9.18	0.68
Diastolic blood pressure (mmHg)	71.2 ± 6.4	70.0 ± 6.89	0.24
Total cholesterol (mmol/L)	3.93 ± 0.70	3.90 ± 0.72	0.78
Triglyceride (mmol/L)	1.00 ± 0.29	2.03 ± 0.62	<.001
High density lipoprotein cholesterol (mmol/L)	1.24 ± 0.25	0.94 ± 0.16	<.001
Glucose (mmol/L)	4.06 ± 0.55	3.87 ± 0.78	0.09
Sex			
Male	93 (50.3)	36 (60.0)	0.42
Female	92 (49.7)	24 (40.0)	

TG: triglyceride, HDL-C: high density lipoprotein cholesterol

^a^ The cut-off point for high and low TG/HDL-C ratio was 1.04 for boys and 0.81 for girls, according to the sex-specific 75th percentiles for TG/HDL-C ratio in the Korean population at age of 16 years

Table 3 shows the results of the multiple regression analyses. After about 20 years of follow-up, a total of 21 participants experienced hypertension at adulthood. A higher prevalence of hypertension was observed in the high TG/HDL-C group than in the low TG/HDL-C group (18.3 vs. 5.4%). The unadjusted odds ratio for hypertension in the high TG/HDL-C ratio group, compared with the low TG/HDL-C ratio group, was 3.93 (95% confidence interval 1.58–9.79). After adjusting for sex and age at follow-up, the odds ratio was 3.62 (95% confidence interval 1.43–9.17). Additionally, after adjusting for baseline SBP, waist circumference, total cholesterol, and fasting glucose, the odds ratio was 3.40 (95% confidence interval 1.24–9.31).

**Table 3:** Association between adolescent TG/HDL-C ratio and adult hypertension

	Total	Adult hypertension (%)	Model 1^a^	Model 2^b^	Model 3^c^
TG/HDL-C group^d^					
Low	185	10 (5.4)	1.00 (ref)	1.00 (ref)	1.00 (ref)
High	60	11 (18.3)	3.93 (1.58–9.79)	3.62 (1.43–9.17)	3.40 (1.24–9.31)

TG: triglyceride, HDL-C: high density lipoprotein cholesterol

Data are presented as odds ratio (95% confidence interval)

^a^Model 1: Unadjusted

^b^Model 2: Adjusted for sex and age at follow-up

^c^Model 3: Adjusted for sex, age at follow-up, adolescent systolic blood pressure, waist circumference, total cholesterol, and fasting glucose

^d^The cut-off point for high and low TG/HDL-C ratio was 1.04 for boys and 0.81 for girls, according to the sex-specific 75th percentiles for TG/HDL-C ratio in the Korean population at age of 16 years

## Discussion

We analysed 245 adolescents from the age of 16 years until adulthood. After a follow-up of about 20 years, adolescents in the high TG/HDL-C ratio group showed a higher prevalence of hypertension in early adulthood than those adolescents in the low TG/HDL-C ratio group. The risk for hypertension was about 3-fold higher in the high TG/HDL-C group, and the association was statistically significant, after controlling major confounders.

The TG/HDL-C ratio is a simple and useful index to identify apparently healthy individuals who are insulin resistant and at increased cardiometabolic risk [10–12]. High TG/HDL-C ratio is associated with metabolic syndrome [13], increased arterial stiffness [14], diabetes, and coronary heart disease [11, 15]. The ability of the TG/HDL-C ratio to predict mortality from coronary heart disease or cardiovascular diseases is equivalent to or better than that of metabolic syndromes [15]. Similar associations between TG/HDL-C ratio and cardiovascular risks have been observed in children and adolescents [16, 17]. Body mass index and waist circumferences were higher in adolescents with higher TG/HDL-C ratios [8, 18], and adolescents in the highest TG/HDL-C ratio tertile had the stiffest vessels, as measured by brachial distensibility, augmentation index, and carotid-femoral pulse-wave velocity [8].

While the TG/HDL-C ratio has been reported to be associated with insulin resistance and cardiometabolic risks, relatively few studies have reported the association between TG/HDL-C ratios and blood pressure or hypertension. One study followed up 5971 middle eastern women for about 6.4 years and reported that higher TG/HDL-C ratios were associated with significantly higher risks of hypertension [7]. In this study, the highest TG/HDL-C ratio quartile group had a 1.7-fold higher risk of hypertension than the lowest quartile. This study also compared the predictability of hypertension by several types of lipid measurements. TG, HDL-C, and TG/HDL-C ratio were the strongest predictors of hypertension. Similar results have also been found from studies on adolescents [8, 17, 19]. In a cross-sectional study of adolescents, the highest TG/HDL-C ratio tertile group had higher SBP, DBP, and arterial stiffness levels [8]. TG/HDL-C ratios positively correlated with systolic, diastolic, and mean blood pressure in 67 obese children, who were 6–12 years of age [17]. SBP and DBP significantly increased from the lowest to the highest TG/HDL-C ratio quantiles in 884 children between the ages of 6 and 16 years [17, 19].

Previously, we reported elsewhere that serum lipid levels in adolescent can predict dyslipidaemia in adulthood using the Kangwha Study data [20]. Our data support that measurement of serum lipids in an individual’s early lifetime, could be useful to predict dyslipidaemia and also hypertension. Other previous studies have reported the predictability of cardiovascular diseases or related risk factors, including metabolic syndromes and atherosclerosis by using serum lipid levels [21–23]. We could assess future cardiovascular risk by measuring serum lipids in early lifetime. However, this issue should be carefully considered from multiple perspectives, including cost, benefit, and the harm of taking blood samples from healthy children and adolescents. According to the United States Preventive Services Task Force, the current evidence is insufficient to assess the usefulness of screening for lipid disorders in children and adolescents [24, 25].

The major strength of this study is that we followed up our study participants over a long duration, from adolescence to adulthood. Among the many studies that have evaluated the association between lipid profiles and cardiovascular diseases and risk factors, this study may be the only one to observe participants ranging from adolescents to early adults. There are also several limitations to this study. First, the sample size was small, and many participants were lost to follow up. Participants who completed the follow-up had a higher baseline height than those who did not, but there were no other significant differences in baseline characteristics between these groups (Additional file 1: Table S2). These findings support the idea that the follow-up was not affected by baseline TG/HDL-C ratios. The participants were residents on a rural island in Korea; therefore, the generalizability is limited. Further prospective studies that include urban populations or participants from other countries, are needed. Second, the method of blood pressure measurement changed between baseline and follow-up examinations. However, all examinations were conducted by investigators trained with a standardised measurement protocol to minimize the measurement error. Third, we did not assess the exact age at baseline, because the date of examination was not recorded. There could be residual confounding factors; however, as the participants were in the same grade at baseline, the variation of the baseline age would be small.

## Conclusions

In conclusion, adolescents with high TG/HDL-C ratios are at risk for future hypertension in early adulthood. Further evidence is required to support this finding and to evaluate whether to introduce screening tests early in an individual’s lifetime.

## Additional file

Additional file 1: **Table S1.** Reference value (sex-height-specific 95th percentile of blood pressures) for adolescent hypertension. **Table S2.** Baseline characteristics of the participants according to follow-up. **Table S3.** Multivariable logistic regression analysis for cholesterol at adolescence with adult hypertension. **Table S4.** Multivariable logistic regression analysis for cholesterol at adolescence with adult hypertension. **Table S5.** Multivariable linear regression analysis between cholesterol and blood pressure. **Table S6.** Sensitivity analysis after excluding participants who were followed-up via online or mail surveys. (DOCX 26 kb)

## Abbreviations

BMI: Body mass index
DBP: Diastolic blood pressure
HDL-C: High density lipoprotein cholesterol
SBP: Systolic blood pressure
TG: Triglyceride
