# Supplementary material for: Triglyceride to high density lipoprotein cholesterol ratio among adolescents is associated with adult hypertension: the Kangwha study
Source: Lipids Health Dis. 2018 Sep 10;17:212. doi: 10.1186/s12944-018-0861-y (PMC6131866; doi:10.1186/s12944-018-0861-y)
Supplement: Supplementary file 1 — Table S1. Reference value (sex-height-specific 95th percentile of blood pressures) for adolescent hypertension. Table S2. Baseline characteristics of the participants according to follow-up. Table S3. Multivariable logistic regression analysis for cholesterol at adolescence with adult hypertension. Table S4. Multivariable logistic regression analysis for cholesterol at adolescence with adult hypertension. Table S5. Multivariable linear regression analysis between cholesterol and blood pressure. Table S6. Sensitivity analysis after excluding participants who were followed-up via online or mail surveys. (DOCX 26 kb) [file 12944_2018_861_MOESM1_ESM.docx]

Additional file 1

Table S1. Reference value (sex-height-specific 95th percentile of blood pressures) for adolescent hypertension

|  | Systolic blood pressure (mmHg) | Diastolic blood pressure (mmHg) |
| --- | --- | --- |
| Boy |  |  |
| Height (cm) |  |  |
| 0 - 168 | 137 | 75 |
| 168.0 - 175.5 | 139 | 75 |
| 175.6 - | 141 | 76 |
| Girl |  |  |
| Height (cm) |  |  |
| 0 - 156.4 | 126 | 74 |
| 156.5 - 163.5 | 127 | 75 |
| 163.5 - | 128 | 75 |

Values are the 95th percentiles of blood pressure in the Korean population according to age, sex and height.

Table S2. Baseline characteristics of the participants according to follow-up

|  | Follow-up (272) | Non follow-up (470) | p-value |
| --- | --- | --- | --- |
| Height (cm) | 165.6 ± 8.0 | 164.0 ± 8.5 | 0.01 |
| Weight (kg) | 56.9 ± 8.5 | 56.7 ± 9.6 | 0.73 |
| Body mass index (kg/m^2^) | 20.7 ± 2.5 | 21.0± 2.9 | 0.17 |
| Waist circumference (cm) | 68.6 ± 6.3 | 68.6 ± 7.5 | 0.97 |
| Systolic blood pressure (mmHg) | 115.9 ± 11.4 | 116.1 ± 11.2 | 0.81 |
| Diastolic blood pressure (mmHg) | 72.3 ± 7.8 | 73.2 ± 7.6 | 0.11 |
| Total cholesterol (mmolL) | 3.96 ± 0.71 | 4.02 ± 0.72 | 0.30 |
| Triglyceride (mmol/L) | 1.25 ± 0.59 | 1.21 ± 0.67 | 0.34 |
| HDL cholesterol (mmol/L) | 1.16 ± 0.26 | 1.20 ± 0.26 | 0.13 |
| Fasting glucose (mmol/L) | 4.03 ± 0.60 | 4.09 ± 0.57 | 0.19 |
| TG/HDL-C ratio | 1.17 ± 0.72 | 1.12 ± 0.89 | 0.33 |
| Sex |  |  |  |
| Male | 144 (52.9) | 186 (39.6) | <.001 |
| Female | 128 (47.1) | 284 (60.4) |  |
| Adolescent hypertension^*^ |  |  |  |
| No | 245 (90.1) | 409 (87.0) | 0.26 |
| Yes | 27 (9.9) | 61 (13.0) |  |

TG: triglyceride, HDL-C: high density lipoprotein cholesterol

Values are presented as mean ± standard deviation, or numbers (%)

^*^ Adolescent hypertension is defined as blood pressure above the age-sex-height-specific 95^th^ percentile of the Korean population.

Table S3. Multivariable logistic regression analysis for cholesterol at adolescence with adult hypertension

| Cholesterol | Adjusted^*^ OR (95% CI) |
| --- | --- |
| TG (mmol/L) |  |
| Q1 (0 - 0.84) | 1.00 (ref) |
| Q2 (0.85 - 1.09) | 2.27 (0.38 - 13.50) |
| Q3 (1.10 - 1.54) | 3.05 (0.56 - 16.56) |
| Q4 (1.55 - ) | 3.46 (0.67 - 17.83) |
| HDL-C (mmol/L) |  |
| Q1 (0 - 0.98) | 1.00 (ref) |
| Q2 (0.99 - 1.13) | 1.29 (0.34 - 4.87) |
| Q3 (1.14 - 1.29) | 1.02 (0.27 - 3.83) |
| Q4 (1.30 -) | 0.71 (0.17 - 2.89) |
| TG/HDL-C ratio |  |
| Q1 (0 - 0.68) | 1.00 (ref) |
| Q2 (0.69 - 0.93) | 4.80 (0.51 - 45.54) |
| Q3 (0.94 - 1.43) | 5.36 (0.59 - 48.80) |
| Q4 (1.44 - ) | 12.11 (1.45 - 101.28) |

TG: triglyceride, HDL-C: high density lipoprotein cholesterol, OR: odds ratio, CI: confidence interval

^*^ The model is adjusted for sex, baseline systolic blood pressure, baseline waist circumference, baseline total cholesterol, baseline fasting glucose, and age at follow-up.

Table S4. Multivariable logistic regression analysis for cholesterol at adolescence with adult hypertension

| Cholesterol | Adjusted^*^ OR (95% CI) |
| --- | --- |
| TG (mmol/L) |  |
| Q1-3 (0 – 1.54) | 1.00 (ref) |
| Q4 (1.55 -) | 1.67 (0.60 - 4.63) |
| HDL-C (mmol/L) |  |
| Q1-3 (0 – 1.29) | 1.00 (ref) |
| Q4 (1.30 -) | 0.65 (0.20 - 2.19) |

TG: triglyceride, HDL-C: high density lipoprotein cholesterol, OR: odds ratio, CI: confidence interval

^*^ The model is adjusted for sex, baseline systolic blood pressure, baseline waist circumference, baseline total cholesterol, baseline fasting glucose, and age at follow-up.

^a^ The cutoff point for high and low TG/HDL-C ratio was 1.04 mmol/L for men and 0.81 mmol/L for women, respectively, according to the age-sex-specific 75th percentiles of TG/HDL-C ratio in the Korean population.

Table S5. Multivariable linear regression analysis between cholesterol and blood pressure

|  | Systolic blood pressure (mmHg) | |  | Diastolic blood pressure (mmHg) | |
| --- | --- | --- | --- | --- | --- |
|  | ß | p-value |  | ß | p-value |
| TG (per 1 mmol/L) | 1.64 | 0.15 |  | 1.90 | 0.04 |
| HDL-C (per 1 mmol/L) | -1.27 | 0.64 |  | -1.08 | 0.64 |
| TG/HDL-C ratio (per 1) | 1.11 | 0.22 |  | 1.27 | 0.09 |

TG: triglyceride, HDL-C: high density lipoprotein cholesterol

Table S6. Sensitivity analysis after excluding participants who were followed-up via online or mail surveys

|  | Total | Adult  hypertension (%) | Model 1^a^ | Model 2^b^ | Model 3 |
| --- | --- | --- | --- | --- | --- |
| TG/HDL-C group* |  |  |  |  |  |
| Low | 175 | 10 (5.7) | 1.00 (ref) | 1.00 (ref) | 1.00 (ref) |
| High | 56 | 10 (17.9) | 3.49 (1.37 - 8.89) | 3.20 (1.24 - 8.31) | 3.04 (1.08 - 8.55) |

TG: triglyceride, HDL-C: high density lipoprotein cholesterol

Data are presented as odds ratio (95% confidence interval).

* The cutoff point for high and low TG/HDL-C ratio was 1.04 mmol/L for men and 0.81 mmol/L for women, respectively, according to the age-sex-specific 75th percentiles of TG/HDL-C ratio in the Korean population.

^a^ Model 1: Unadjusted.

^b^ Model 2: Adjusted for sex and age at follow-up.

^c^ Model 3: Adjusted for sex, age at follow-up, adolescent systolic blood pressure, waist circumference, total cholesterol, and fasting glucose.
